# Development and implementation of ISAR, a new synthesis platform for radiopharmaceutical production

**DOI:** 10.1186/s41181-019-0077-0

**Published:** 2019-09-18

**Authors:** Christopher Frank, Georg Winter, Fredrik Rensei, Victor Samper, Allen F. Brooks, Brian G. Hockley, Bradford D. Henderson, Christian Rensch, Peter J. H. Scott

**Affiliations:** 1 GE Healthcare, Oskar-Schlemmer-Str. 11, 80807 Munich, Germany; 2 GE Healthcare, Uppsala, Sweden; 3 GE Additive, Garching near Munich, Germany; 4 Department of Radiology, University of Michigan, 2276 Medical Science Bldg I, SPC 5610, Ann Arbor, MI 48109 USA

**Keywords:** Microfluidics, Lab on a chip, Automation, [^13^N]ammonia, Myocardial perfusion imaging

## Abstract

**Background:**

PET radiopharmaceutical development and the implementation of a production method on a synthesis module is a complex and time-intensive task since new synthesis methods must be adapted to the confines of the synthesis platform in use. Commonly utilized single fluid bus architectures put multiple constraints on synthesis planning and execution, while conventional microfluidic solutions are limited by compatibility at the *macro-to-micro interface*. In this work we introduce the ISAR synthesis platform and custom-tailored fluid paths leveraging up to 70 individually addressable valves on a chip-based consumable. The ISAR synthesis platform replaces traditional stopcock valve manifolds with a fluidic chip that integrates all fluid paths (tubing) and valves into one consumable and enables channel routing without the single fluid bus constraint. ISAR can scale between the macro- (10 mL), meso- (0.5 mL) and micro- (≤0.05 mL) domain seamlessly, addressing the *macro-to-micro interface* challenge and enabling custom tailored fluid circuits for a given application. In this paper we demonstrate proof-of-concept by validating a single chip design to address the challenge of synthesizing multiple batches of [^13^N]NH_3_ for clinical use throughout the workday.

**Results:**

ISAR was installed at an academic PET Center and used to manufacture [^13^N]NH_3_ in > 96% radiochemical yield. Up to 9 batches were manufactured with a single consumable chip having parallel paths without the need to open the hot-cell. Quality control testing confirmed the ISAR-based [^13^N]NH_3_ met existing clinical release specifications, and utility was demonstrated by imaging a rodent with [^13^N]NH_3_ produced on ISAR.

**Conclusions:**

ISAR represents a new paradigm in radiopharmaceutical production. Through a new system architecture, ISAR integrates the principles of microfluidics with the standard volumes and consumables established in PET Centers all over the world. Proof-of-concept has been demonstrated through validation of a chip design for the synthesis of [^13^N]NH_3_ suitable for clinical use.

## Background

Positron emission tomography (PET) is a functional molecular imaging technique that utilizes bioactive molecules labeled with a positron-emitting radionuclide (radiopharmaceuticals) to non-invasively quantify physiological and biochemical processes (for a general review of PET imaging, see: Ametamey et al. 2008). During the PET scan, subjects receive a radiopharmaceutical dose and the scanner detects pairs of coincident 511 keV gamma photons emitted indirectly by the PET radionuclide. Data is then reconstructed to generate the PET image. Several PET radiopharmaceuticals have been approved for clinical use (Vavere and Scott 2017) and, reflecting this, millions of PET scans occur worldwide every year (PET Imaging Market Summary Report 2018). The short half-life of PET radionuclides (typically minutes – hours) mandates that radiopharmaceuticals are manufactured at a radiopharmacy facility that is in close proximity to the clinical PET scanner(s) on a daily basis. The synthesis and delivery of PET radiopharmaceuticals for clinical use present some unique challenges including the need for a manufacturing process that is rapid, reliable, automated and affordable so as to be compatible with short-lived PET radionuclides, current Good Manufacturing Practice (cGMP), radiation safety and healthcare economics. To accomplish daily dose production, radiopharmaceuticals are typically manufactured using automated synthesis modules housed in lead-lined hot-cells (Thompson and Scott 2019).

**Fig. 1:**
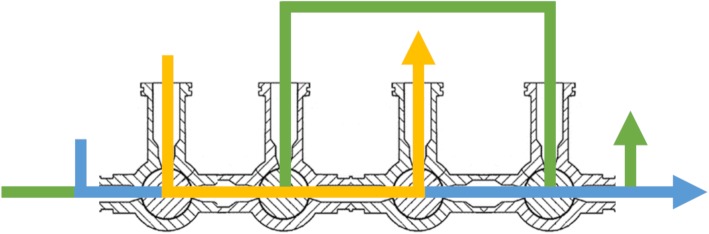
Cross section of single fluid bus consisting of a typical row of stopcock valves – the colored lines represent different fluid paths that cross each other and therefore will contaminate each other with residual liquid

Many traditional radiochemistry synthesis modules use a single fluid bus architecture in conjunction with stopcock valve manifold(s), in which every fluid runs through parts of the central pathway (Fig. 1). They are cassette-based systems that require a varying degree of customer assembly, optimized for a single radionuclide and designed to produce one or a few large batches (e.g. tens to hundreds of GBq) of a given PET radiopharmaceutical for routine clinical use. For example, because [^18^F]fludeoxyglucose ([^18^F]FDG) is the most common PET radiopharmaceutical in clinical use and the half-life of ^18^F (t_1/2_ = 110 min) is compatible with centralized production, many synthesis modules have been designed for the express purpose of manufacturing [^18^F]FDG (Ametamey et al. 2008; Yu 2006).

Therefore, the implementation of other syntheses requires subsequent adaptations of the [^18^F]FDG baseline architecture (Yu 2006). However, the synthesis and utilization of all PET radiopharmaceuticals does not necessarily fit this manufacturing paradigm. For example, in developing markets there is a tendency to synthesize single patient doses as needed, rather than large multidose batches. Alternatively, the use of short-lived PET radionuclides (e.g. ^15^O (t_1/2_ = 2 min), ^13^N (t_1/2_ = 10 min), ^11^C (t_1/2_ = 20 min), ^68^Ga (t_1/2_ = 68 min)) to label other radiopharmaceuticals necessitates de-centralized production of different radiopharmaceuticals in batches suitable for 1–2 patients at a time on a continuous basis throughout the day (Wang et al. 2010; Elsinga 2012; Keng et al. 2012a; Rensch et al. 2013). Production of several batches on a single fluid path architecture relies on replacing consumables multiple times per day, or rinsing procedures between production steps (to avoid cross contamination), which creates further expense and/or complexity in validation of these cleaning processes (Haka et al. 2017).

There is a need for a more flexible radiopharmaceutical synthesis platform that is compatible with different levels of radioactivity (single dose-on-demand production or multidose batches) as well as a variety of radionuclides and/or radiopharmaceuticals, and which can fit into the space confines of a typical radiopharmacy laboratory with only one or two hot-cells. Ideally, it does not rely on cleaning procedures and aligns with the state-of-the art approach of single-use disposables. To date, efforts in this direction have focused on development of microfluidic approaches for radiopharmaceutical manufacturing (Amaraesekera et al. 2013; Audrain 2007; Awasthi et al. 2014; Bejot et al. 2010; Bouvet et al. 2011; Bouvet et al. 2012; Bouvet and Wuest 2013; Chen et al. 2014; Collier et al. 2010, 2017; De Leonardis et al. 2010, 2011; Gaja et al. 2012; Gillies et al. 2006; Lu and Pike 2007; Lu and Pike 2010; Elizarov 2009; Elizarov et al. 2010; Fortt and Gee 2013; Kealey et al. 2011; Keng et al. 2012b; Keng and van Dam 2015; Lee et al. 2005; Liow et al. 2005; Liu et al. 2011; Liu et al. 2013; Lu et al. 2004, 2009, 2010; Matesic et al. 2017; Miller 2009; Miller et al. 2010, 2011; Pascali et al. 2010, 2011, 2013; Pascali and Salvadori 2016; Rensch et al. 2012, 2013; Selivanova et al. 2012; Simms et al. 2012; Steel et al. 2007; Ungersboeck et al. 2011, 2012a, 2012b; Voccia et al. 2009; Wang et al. 2010; Wang et al. 2019; Wester et al. 2009; Wheeler et al. 2010; Yokell et al. 2012; Zeng et al. 2013), as well as purification/reformulation (Chao et al. 2017) and quality control (QC) testing (Ha et al. 2017; Taggart et al. 2016; Ly et al. 2018). While the benefits of microreactors for radiopharmaceutical synthesis have been well documented in this multitude of literature precedent, and they have been used to prepare radiopharmaceuticals for clinical use (Lebedev et al. 2013; Liang et al. 2014a, 2014b; Rensch et al. 2014), there are continuing challenges that have prevented them from commercialization and really transitioning into widespread use to date (Chiu et al. 2017). Notable exceptions in the PET space include ABTs BG75 (Awasthi et al. 2014) and the Advion Nanotek (Pascali et al. 2014), but both systems remain confined to only a small number of laboratories around the world as of 2019.

The reasons for the slow uptake of microfluidic systems within the PET community despite the well documented benefits are numerous and depend on the particular application objective, but from the perspective of a clinical end user, include: I) system cost (in our experience, some commercial microfluidic systems are almost double the cost of standard radiopharmaceutical synthesis modules), II) the lack of availability of affordable components due to dependence on chips, III) issues with the *macro-to-micro interface,* or general incompatibility between microfluidic volumes (≤ 0.05 mL) and the typical “large-scale” volumes used in cyclotrons and radiopharmaceutical dosing (≥ 10 mL), and IV) a lack of improvement over existing technology.

**Fig. 2:**
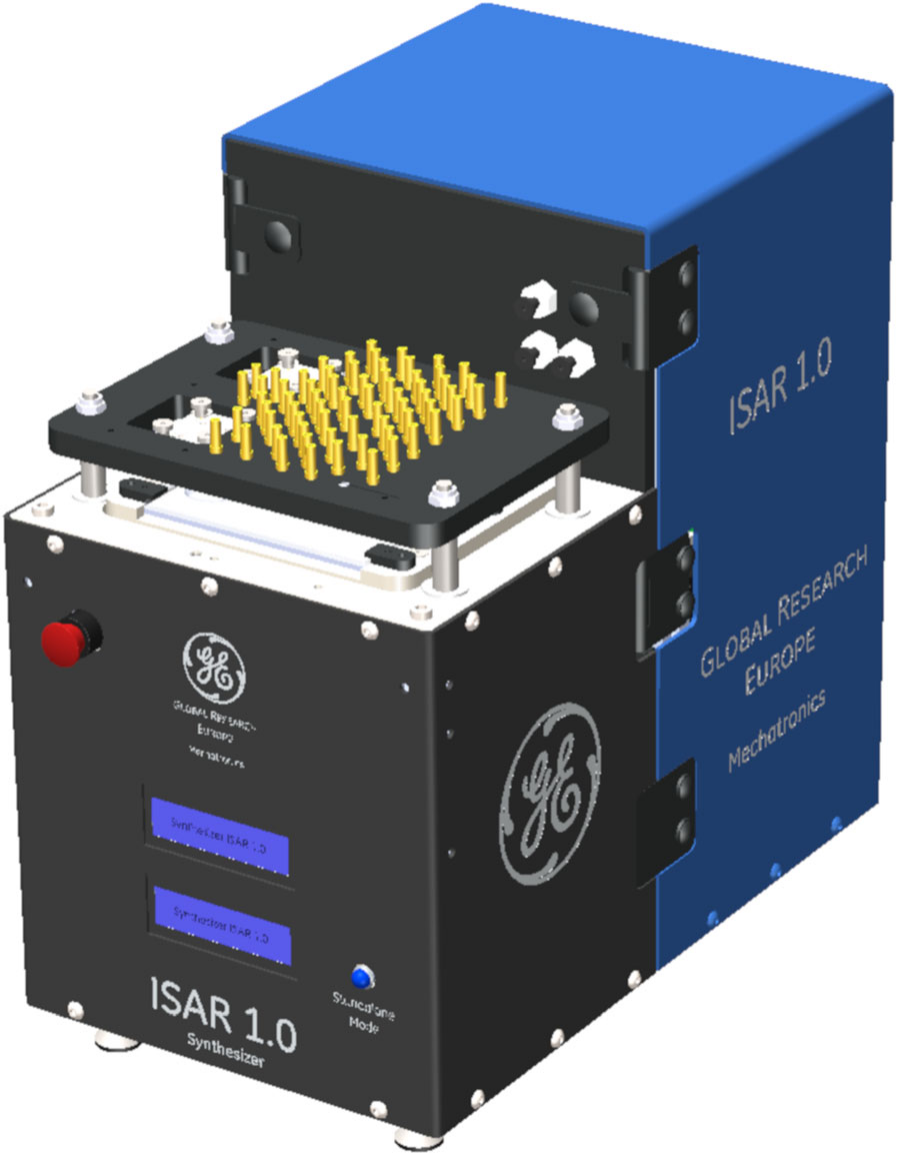
ISAR unit (W x L x H 20 × 34 × 34 cm) with 70 luer connectors on top enabling the *macro-to-micro interface* between the fluidic chip and standard laboratory items used in the production of radiopharmaceuticals

**Fig. 3:**
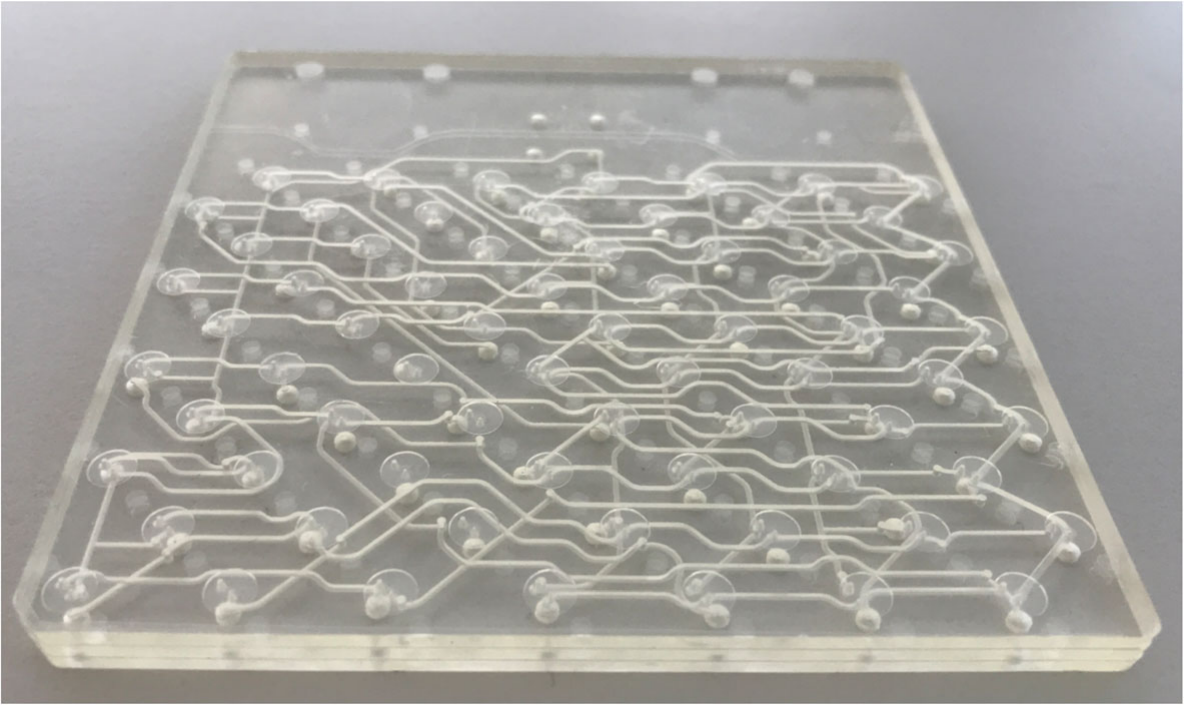
ISAR chip (W x L x H 10,4 × 10,4 × 0,6 cm) with 70 on-chip membrane valves hosting multiple parallel fluid paths enabling point-to-point routing – the shown layout is designed for nine production runs of [^13^N]NH_3_

**Fig. 4:**
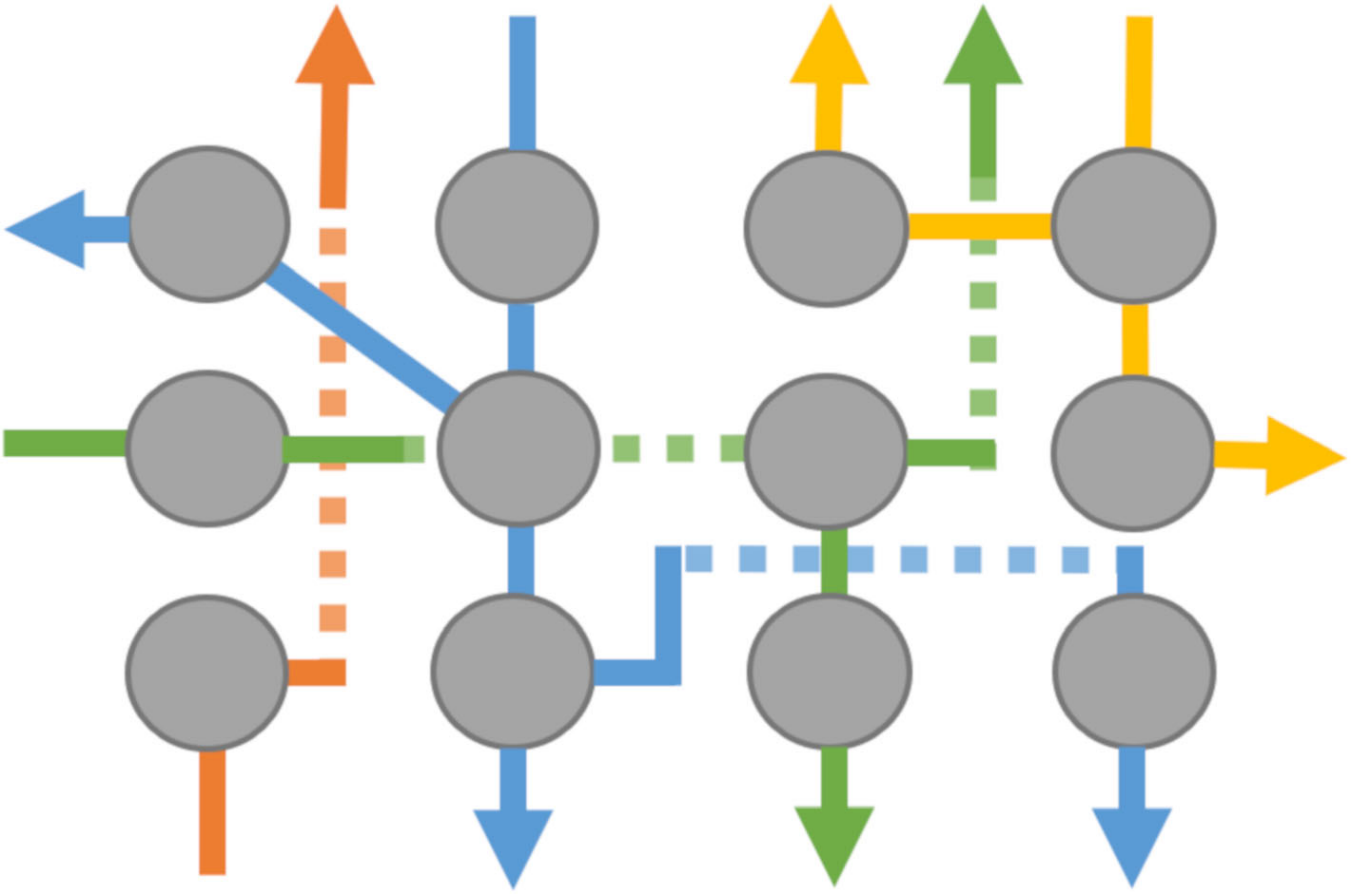
Illustration of the *parallel fluidic architecture* running on both sides of the same layer (doted lines on the opposite side), thus enabling contamination free crossing of fluid paths

To address a number of these issues, in this report we introduce ISAR^1^ (Fig. 2) as well as the ISAR chip (Fig. 3), a readily available injection molded component that is entirely manufactured out of cyclic olefin copolymer (COC) and integrates all reaction vessels, fluid paths (tubing) and valves into one consumable, and enables direct point-to-point channel routing. This work reduces to practice the lab-on-chip concepts that GE have been developing in recent years (for example, see: Rensch et al. 2014, 2017, 2018). Such a concept is analogous to multi-layered printed circuit boards in electronics. This configuration eliminates a single fluid bus, thereby reducing challenges associated with cross-contamination and the need for complex cleaning validation (Haka et al. 2017). While the multi-layer fluid routing facilitates complex syntheses, the applicability to multiple independent productions has also been realized, and is the focus of this paper. The ISAR system offers both *parallel fluidics* (Frank et al. 2018) and *independent* fluidics (this work) (Fig. 4), potentially disruptive technologies that overcome the single fluid bus and associated challenges at the *macro-to-micro interface* by marrying standard volumes, techniques, and off-the-shelf components for radiopharmaceutical synthesis with the established benefits of a microreactor setup.

**Fig. 5:**
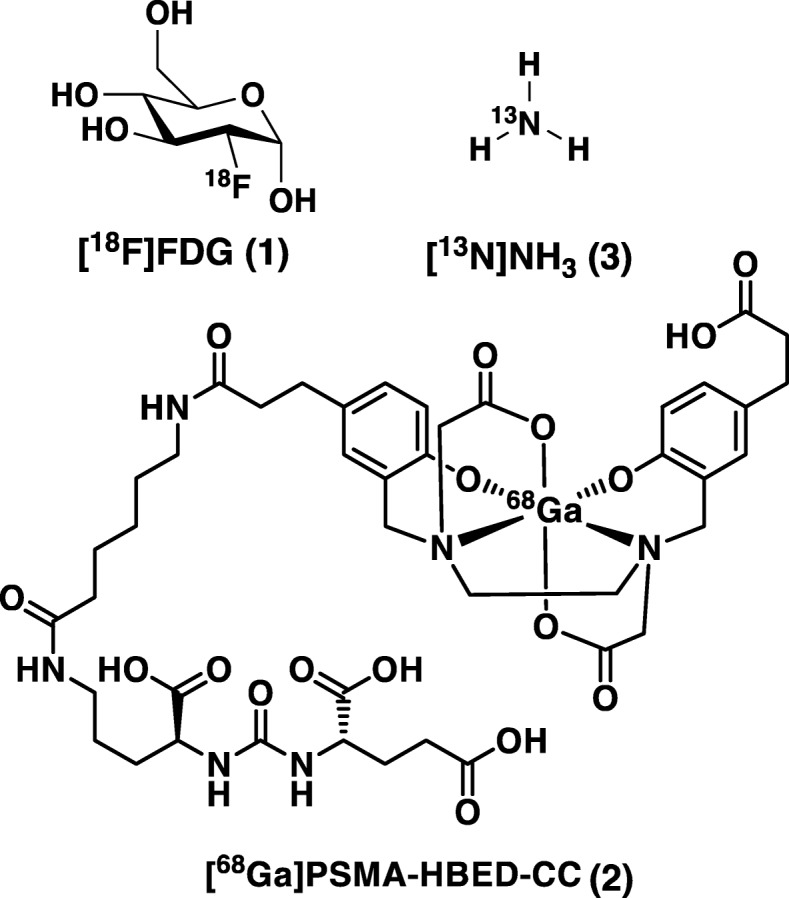
The different Isotopes used, and radiopharmaceuticals produced with ISAR so far – (1) [^18^F]fludeoxyglucose; (2) [^68^Ga]Ga-PSMA-HBED-CC; (3) [^13^N]NH_3_

Preliminary proof-of-concept was recently shown through the on chip synthesis of two commonly used PET radiopharmaceuticals (Fig. 5). [^18^F] FDG (**1**) was produced with an overall activity yield (AY) of ~ 65% (> 100 GBq) in less than 25 min and [^68^Ga]PSMA-HBED-CC (**2**) was manufactured in a dual run scenario (two separate runs from the same consumable) each resulting in ~ 44% AY (< 11.5 min synthesis time) (Frank et al. 2018). On our development roadmap, these two steps confirmed I) fundamental technical feasibility of ISAR, process efficiencies competitive to state-of-the-art routine [^18^F] FDG production, II) reduced synthesis time, and III) capability of multi-run architectures for e.g. two ^68^Ga syntheses from a single set of reagents. Following successful proof-of-concept demonstration, we next sought to install ISAR under real-life conditions at a busy PET center and compare performance with existing in-house standards for a challenging process. Concurrent with these efforts, we realized that parallel processing is also well suited for multi-run schemes of simple production processes, such as [^13^N]NH_3_ for myocardial perfusion imaging. We therefore selected synthesis of [^13^N]NH_3_ for high resolution myocardial perfusion imaging (MPI) with which to conduct the first field test of ISAR. Validation of the synthesis of [^18^F] FDG and [^68^Ga] PSMA for clinical use are also ongoing and will be reported in due course.

Historically, assessments of myocardial perfusion are conducted with ^82^Rb because the radionuclide is readily available from generators (Yoshinaga et al. 2010; Nakazato et al. 2013). However, owing to generator supply challenges and reduced imaging quality stemming from the relatively high positron energy of ^82^Rb, there is motivation to conduct high resolution myocardial perfusion imaging (MPI) with cyclotron-based [^13^N]NH_3_ instead (Murthy et al. 2016). The transition to MPI with [^13^N]NH_3_ presents significant production challenges to PET radiopharmaceutical manufacturers however. Specifically, the short half-life of ^13^N (t_1/2_ = 10 min) requires continuous production of high numbers of doses throughout the day to meet busy imaging schedules. For particularly short-lived radionuclides, the regulations allow quality control (QC) testing to be conducted on an initial sub-batch of the radiopharmaceutical (< 823 > Positron Emission Tomography Drugs for Compounding, Investigational, and Research Uses, in USP 42-NF 37, 2018). Subsequent sub-batches can then be manufactured and sent directly to the imaging suite in a given period (usually 24 h) without additional QC testing. Despite the convenient sub-batch allowance, the traditional Devarda’s alloy method used for routine synthesis of [^13^N]NH_3_ at the University of Michigan PET Center requires new reagents and consumables for production of each sub-batch (Scott 2012). This has inherent costs, operational complexity (sub-batches need to be manufactured and purified from target impurities rapidly given the short half-life; high numbers of batches are required per day (1–2 per patient); space constraints limit how many batches can be set up in advance using a single hot-cell; [^13^N]NH_3_ production needs to fit into a busy workflow where multiple lots of multiple PET radiopharmaceuticals are already being prepared simultaneously), and undesirable radiation exposure to workers who have to replace spent consumables throughout the day that contain residual nitrogen-13 in spite of the short half-life. As such, we had a strong interest in being able to prepare multiple 5.6–7.4 GBq sub-batches of [^13^N]NH_3_ with a single consumable so that, after purification and transport to the imaging suite, it is possible to draw both rest (370 MBq) and stress (740 MBq) doses 20 min apart from a single batch. Leveraging the parallel fluidic architecture of our new chips as well as the small dead volumes, in this paper we report installation of ISAR at the University of Michigan PET Center and use of the system to produce up to 9 doses of [^13^N]NH_3_ (Fig. 5 (**3**)) using a single consumable chip and set of reagents.

## Methods

### Safety and hazard considerations

Radioactivity and all hazardous chemicals were used by trained personnel under the supervision of Environmental Health and Safety and the Radiation Policy Committee at the University of Michigan.

### Chip Design and manufacturing

**Fig. 6:**
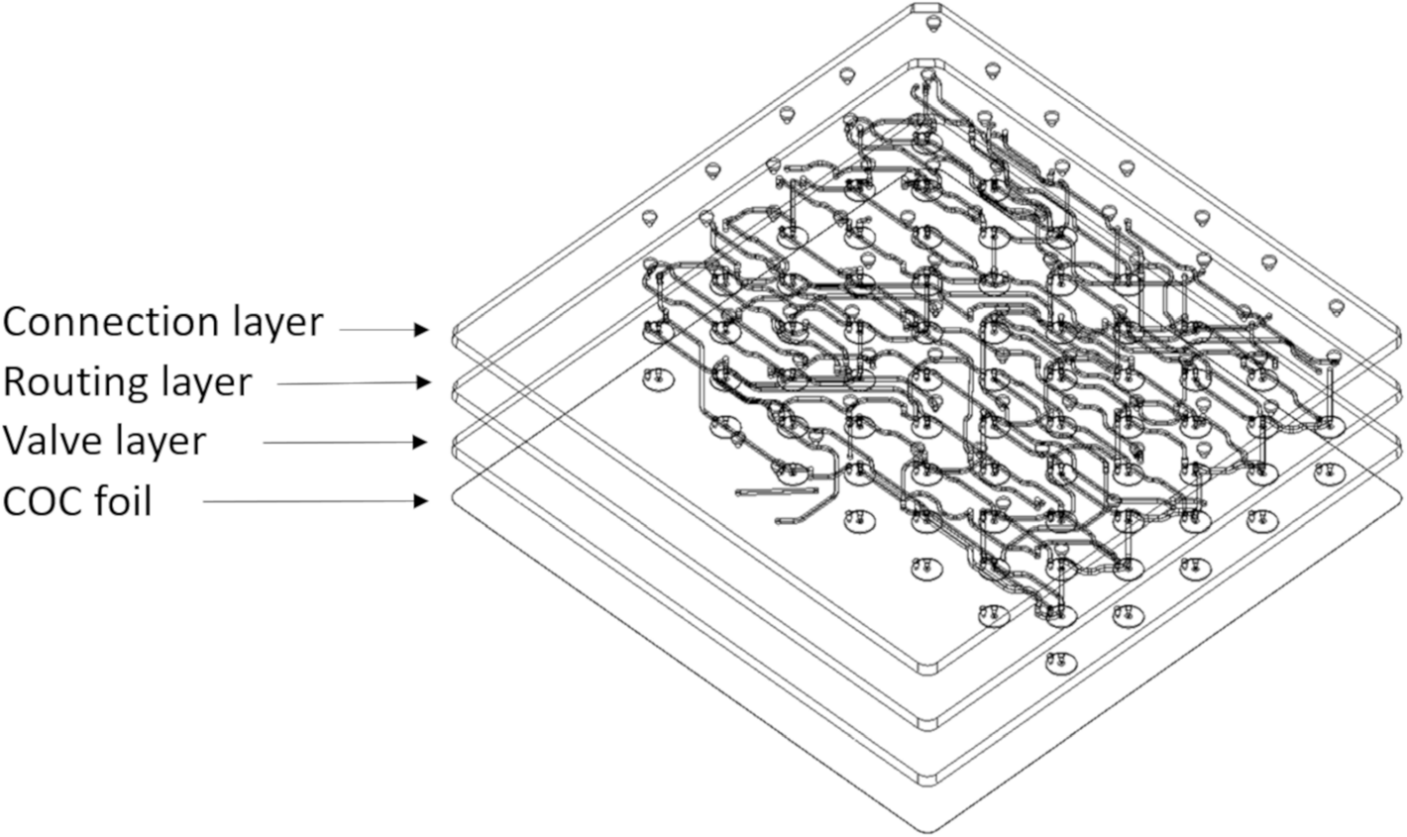
The different parts of the ISAR chip. The three different layers and the foil combined into a complete chip during the bonding process

The fluidic chip consumable integrates all reaction vessels, fluid paths and valves into one consumable and is a readily available injection molded component that is entirely manufactured out of cyclic olefin copolymer (COC). This material offers excellent resistance against acids, bases, and solvents commonly used in the production of radiopharmaceuticals (e.g. acetonitrile and dimethyl sulfoxide). Three layers and a COC foil are joined in a bonding process without the use of any adhesives or binders, reducing material impurities and unwanted residuals (Fig. 6). To date over 1500 chips have been produced with a scrap rate below 1%. Representative samples from each batch are checked under the microscope and we have not observed any wear in the manufacturing tools. Bond quality is currently conducted by visual inspection and destructive testing of samples.

The COC foil forms the valves together with the valve layer that contains 70 membrane valves. Those valves are connected through the routing layer towards reaction structures on the chip, thus replacing the majority of tubing utilized in a conventional consumable. At the same time, the available routing logic is increased by the high packing density of channels and structures on the chip. The valve array pattern is dictated by injection molding considerations and the connection points follow an equal distance distribution with two exceptions: two positioning pins and an additional area for further connection points between the two reaction chambers. The fluid paths are then designed to fit together with both patterns. The final chip dimensions are 104 × 104 × 6 mm (with a 3 mm 45° recess at one corner to help with correct orientation when used) and valve spacing is 10.3 mm. Valves, as the critical structures, are specified to function after > 100 actuation cycles and at 5 bar (72.5 psi) over pressure. To test this, > 250 valves were evaluated as shown in Table 1.

**Table 1:** Valves Used in ISAR Chips

Entry	Test	Result	n
1	N_2_ (2 bar) leakage	< 10 μL/min	> 250
2	Valve functional after > 100 cycles	Pass	> 250

**Fig. 7:**
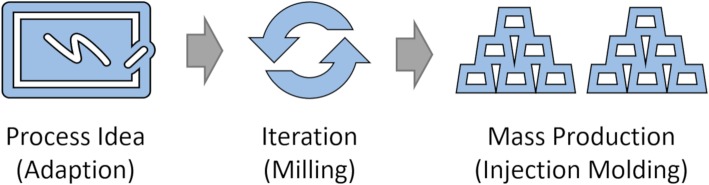
Illustration of the chip design process. An existing chip layout is adopted to a new process or the layout is created from scratch. Iterations of the layout are fabricated by milling the layout into a blank routing layer, which later can be transferred to injection molding for mass production

Access to off-the-shelf components such as solid phase extraction (SPE) cartridges and fluid vials is maintained through 70 connection points on the connection layer, that can serve as in-and/or outlets. The valve and connection layers are standardized and injection molded parts. The routing layer can be manufactured by rapid prototyping (milling of a blank), or injection molding during commercialization ramp-up. These are reliable techniques for manufacturing microfluidic chips (Attia et al. 2009; Guckenberger et al. 2015), and have been successfully demonstrated for [^18^F] FDG and [^13^N]NH_3_ ISAR chips. This enables manufacturing scalability from prototyping across low rate initial production to cost competitive mass production of chips. In addition, iterative process design is possible by making adaptive changes to the routing layer in the prototyping phase. It allows the production of custom-made chips in the scale of a small series (10 to 500 copies) at acceptable cost. If the number of chips required is high enough to justify it, the finalized design can then be transferred to injection molding (Fig. 7).

The routing logic contains different reaction structures and channels customized towards the process at hand. It can make use of two reaction chambers (~ 650 μL each) and a variety of different fluidic structures, designed for mixing, metering of volumes, and dilution. Everything can be either directly routed point-to-point (preventing cross contamination or the need for cleaning validation), in a row, or in a combination of both. External components such as standard SPE cartridges or vials can be routed according to the same principle across I/Os of the connection layer. The channel diameters can be selectively scaled from micro (< 50 μm) to macro (> 2 mm) depending on the liquid volumes that need to be transferred and optimized (e.g. fluid volume, mass transfer, heat transfer, fluid viscosity and/or residual dead volume). The developer can start with a sub-optimal design for first results and (re) iterate as required.

### ISAR hardware

**Fig. 8:**
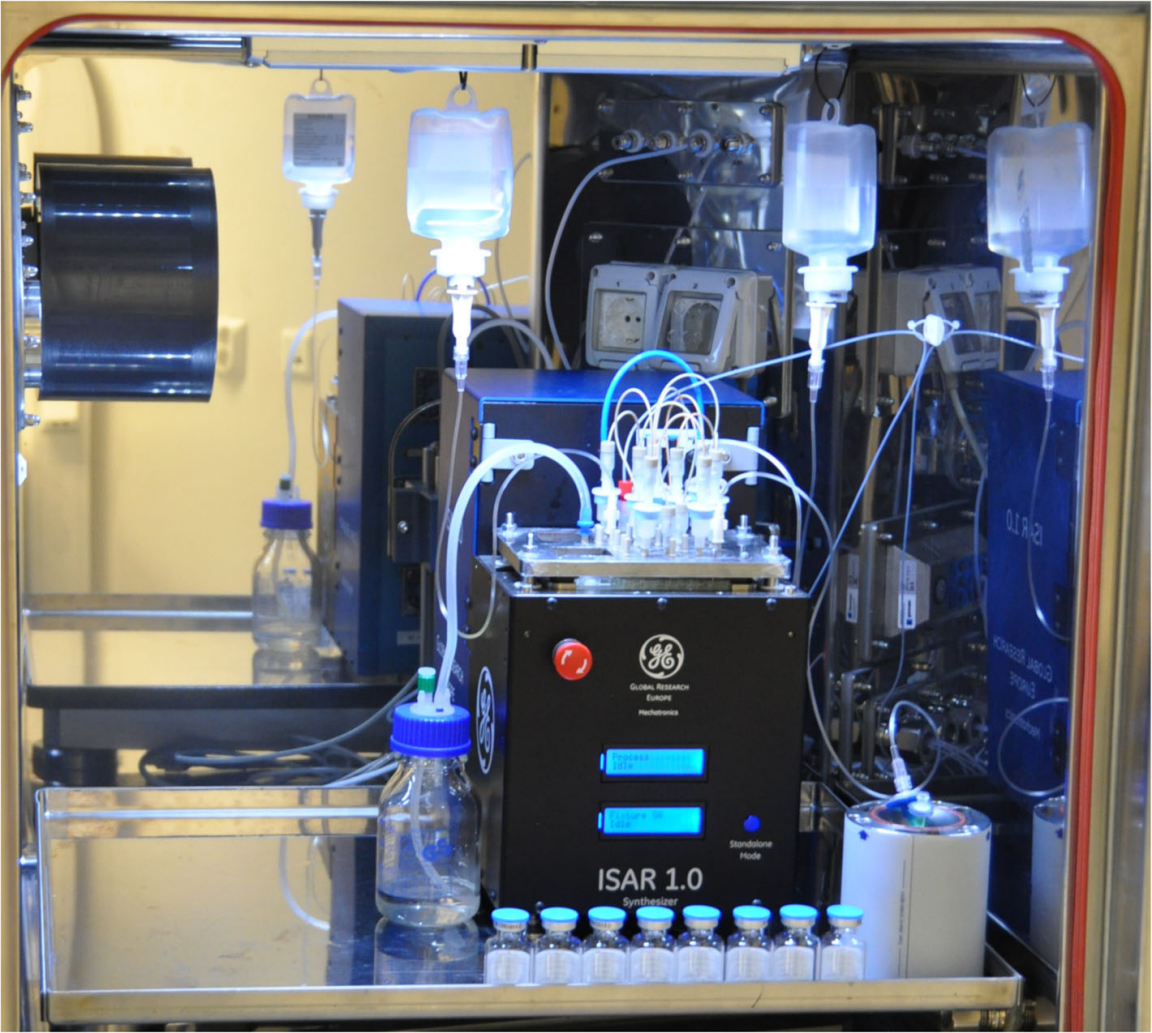
ISAR unit installed in hot-cell similar to the set up at the University of Michigan PET Center. Installation at the University involved placing the ISAR module in a hot-cell, connecting it to mains power, N_2_ gas (6 bar), a control laptop (ethernet) and the delivery line from the [^13^N]NH_3_ target on the cyclotron. Installation was completed in less than 1 h

**Fig. 9:**
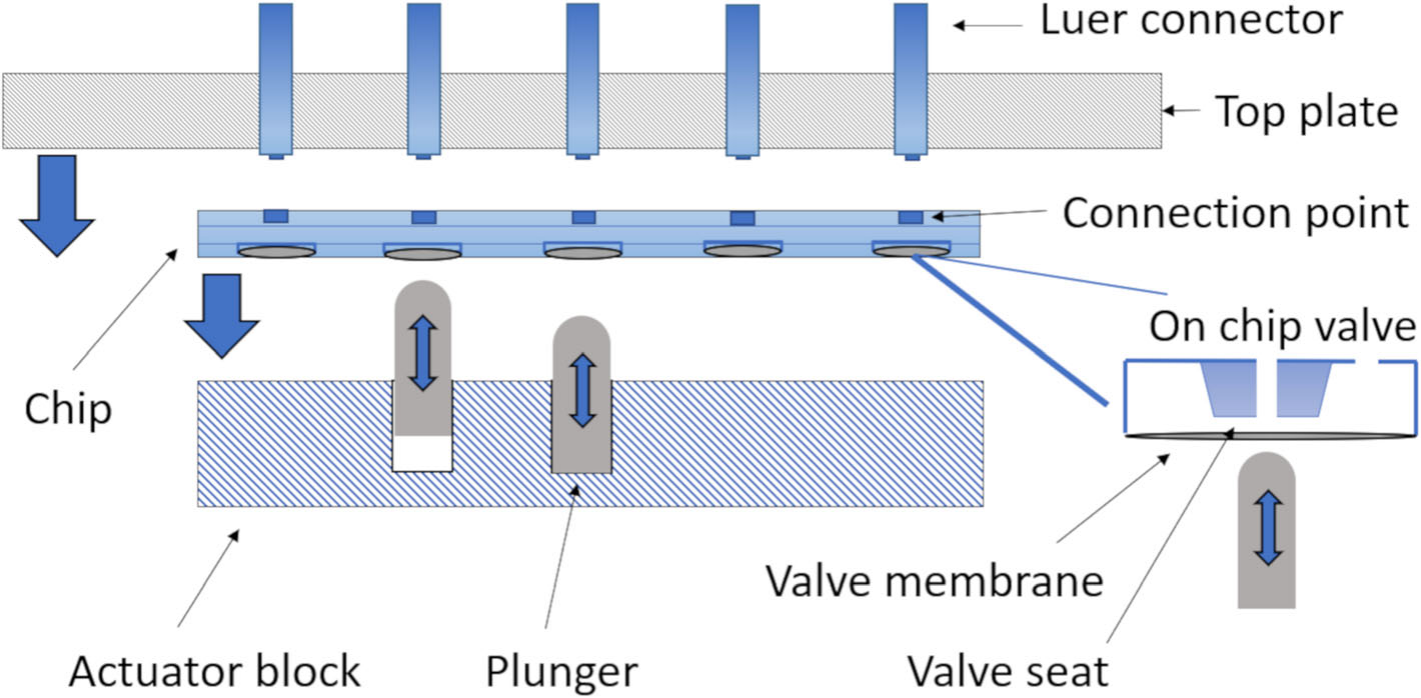
Functionality of chip clamping and on-chip membrane valves. The top plate clamps the chip down against the extended plungers. The plungers press the valve membranes inwards against the valve seats and close therefore the valves are normally closed. In the same clamping motion, the luer connectors are joined together with the connection points on the chip

**Fig. 10:**
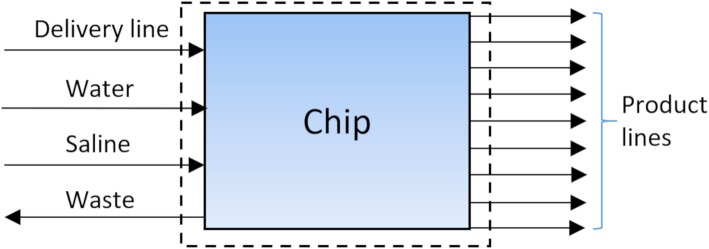
Simplified system boundaries for the nine-run [^13^N]NH_3_ chip. The radioactivity enters the chip through the delivery line and is retained on one of nine SPE cartridges on separated fluid paths. The transporting target fluid leaves the system again and flows into the waste. Water is used to flush the SPE cartridge and also flows into the waste. Saline is used to elute and formulate the [^13^N]NH_3_ through one of nine product lines

An ISAR unit is a compact, highly automated platform (Figs. 2 and 8), capable of producing various radiopharmaceuticals using consumable chips that integrate all reaction vessels, fluid paths (tubing) and valves into one consumable (Figs. 3, 6, 9 and 10). The fluid paths are then designed to fit together with both patterns and with certain safety and design considerations based on experience in mind From the point of view of routing fluids, the core functionality of the system is based on an actuator block, which enables an individual binary on-chip valve control. The unit and consumable are brought together by clamping the chip down by the top plate against the actuator block, simultaneously connecting the chip to the luer connectors integrated in the top plate and putting the membrane valves in a normally closed position (Rensch et al. 2018). Each valve membrane is pressed against its corresponding on-chip valve seat by a respective electro-pneumatically actuated mechanical plunger (Fig. 9). The combination of electro-pneumatics and the membrane valves allows for a precise, real-time control of valves. Completion of each actuation is achieved within a reliable, defined time window with actuation times of < 10 ms (closed-open-closed cycle in < 30 ms). This real-time valve switching capability is in marked contrast to traditional stopcock valve manifolds, which rely on rotational motion of valve drivers at actuation times often exceeding 1 s. Real-time valve control enables gas-pressure driven fluid transport at accuracies historically only achievable by syringe pumps (Rensch et al. 2017).

ISAR is designed to perform complete radiosyntheses on chip, as recently demonstrated for [^18^F]FDG and [^68^Ga]PSMA-HBED-CC (see: Frank et al. 2018). As such, the ISAR chip includes two reaction chambers, and heating of these chambers is performed by four aluminum heating blocks. One above and below each reaction chamber. Active cooling capabilities are provided by blowing N_2_ gas through the heating blocks, enabling precise control and acceleration of cool-down ramps. Negative pressure can also be applied to the chip by duty cycle control of a vacuum pump, which can also be closed loop controlled, in combination with one of two installed pressure sensors, to a pre-set pressure value within any structure on the chip. Compared with traditional synthesizers, this allows a more precise application of vacuum, improving the overall process control, e.g. for azeotropic drying procedures on-chip as demonstrated for [^18^F]FDG (Frank et al. 2018).

To facilitate gas pressure driven fluid transport, the N_2_ gas source needed for the clamping of the consumable and valve actuation is also further regulated by a mass flow controller, capable of controlling both flow (up to 1000 mL·min^− 1^) and pressure (up to 2 bar). Combined with metering structures and the precise actuating of valves described, no syringe pumps are needed since fluids on the chip can be driven by overpressure. This allows highly efficient fluid recovery (Table 2), and helps to reduce the cost, size and technical complexity of an ISAR unit. In addition, the available functionality for fluid routing and control is increased beyond what is achievable by some of the largest (2x to 3x of ISAR unit size) stopcock valve and syringe pump-based synthesizers currently available.

**Table 2:** Fluid recovery with ISAR

Liquid	Target			Achieved			n
	time	volume	accuracy	time	volume	Standard deviation	
	min	μL	±μL	min	μL	±μL	
H_2_O	2	6000	200	3	6163	56	5
	2	1500	200	4	1532	49	10
	3	300	50	2	298	3	5
EtOH	2	1500	200	4	1512	69	10
	2	400	50	3	378	22	6
	3	100	50	1	101	4	10
MeCN	3	500	50	2	520	10	5
	3	100	50	1	101	7	10

### Radiosynthesis of [^13^N]NH_3_

To produce [^13^N]NH_3_, a custom chip layout was created utilizing the methods described. It enables nine back-to-back productions without the need to reopen a hot-cell (Fig. 8). Each production line on the chip is separated from one another, while still being fed by one delivery line and a single set of fluid-containers, e.g. water, saline, waste (Fig. 10). The chip combined with the ISAR unit acts as a conductor between everything entering and leaving the chip, while preventing cross contamination through downstream separation of each synthesis fluid path and maintenance of a single direction for fluid propagation. This is enabled by the chip architecture as described and is not feasible on conventional stopcock valve-based synthesizers.

[^13^N]NH_3_ was prepared in target on a GE PETtrace cyclotron by irradiating aqueous ethanol (5 mM) at 40 μA for 10–30 min, according to a published method (Wieland et al. 1991). The resulting [^13^N]NH_3_ was then transferred to the ISAR unit through the delivery line. Subsequently it was trapped on one of 9 parallel Waters CM-Light Sep-Pak cartridges connected to the ISAR chip (Fig. 8). The cartridge was rinsed with sterile water for injection, Ph. Eur. (2 mL), and afterwards [^13^N]NH_3_ was eluted from the cartridge using 0.9% NaCl, Ph. Eur. (2 mL), through a 0.22 μm sterile filter (Millipore Millex GS) into a sterile dose vial (Hollister-Stier). QC testing was completed as previously described (Scott 2012). Processing of [^13^N]NH_3_ by ISAR took 5 min after end-of-bombardment.

### Rodent PET imaging with [^13^N]NH_3_

Rodent PET imaging with [^13^N]NH_3_ was conducted in a female Sprague Dawley rat (321 g) in accordance with the standards set by the Institutional Animal Care and Use Committee at the University of Michigan. The animal was anesthetized (isoflurane), intubated, and positioned in a Concorde MicroPET P4 scanner. Following a transmission scan, the animal was injected (*i.v.* via tail vein catheter as a bolus over 1 min) with [^13^N]NH_3_ (10 MBq) and the heart imaged for 30 min (6 × 5 min frames).

**Fig. 11:**
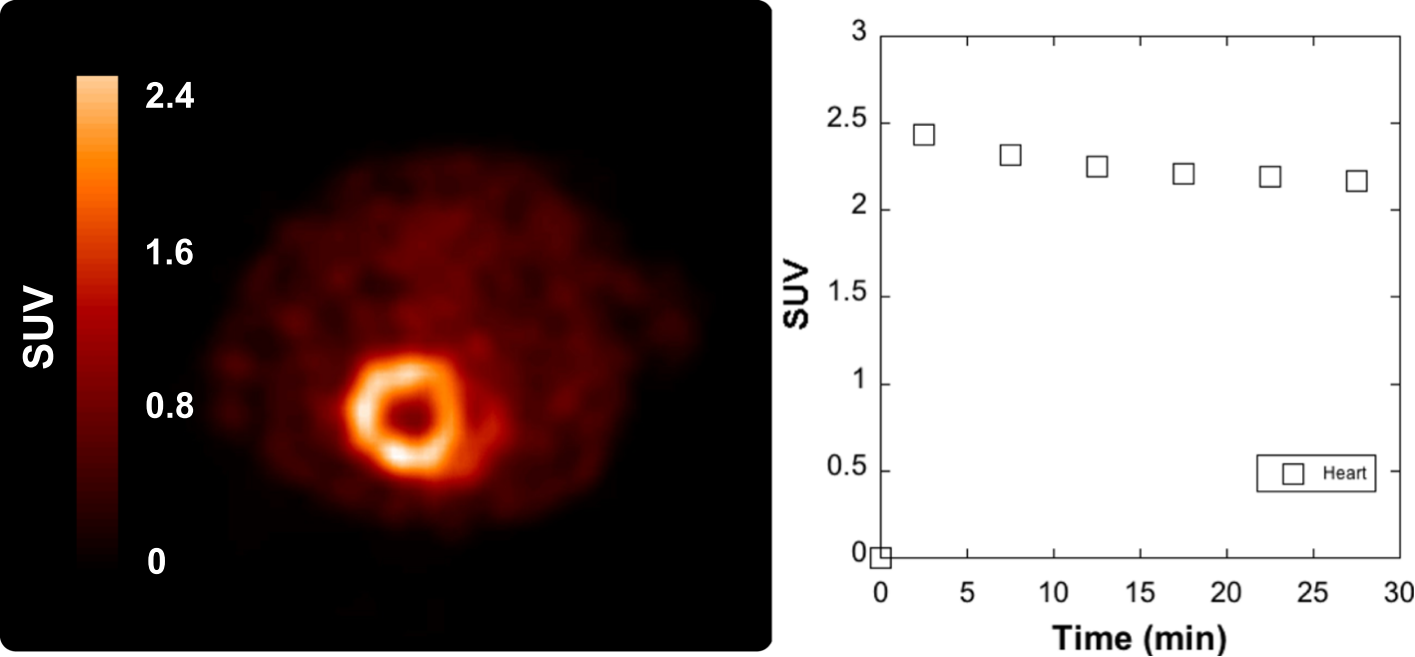
Summed transverse rodent image of [^13^N]NH_3_ (0–30 min after injection of the radiopharmaceutical) showing excellent visualization of the heart and good heart-to background contrast and associated time-radioactivity curve

Emission data were corrected for attenuation/scatter and reconstructed using the 3D maximum a priori method. By using a summed image (Fig. 11), a region-of-interest was drawn over the heart on multiple planes, and the corresponding volume of interest applied to the full dynamic data set to generate a time-activity curve.

## Results and discussion

The ISAR synthesis platform (Figs. 2 and 8) replaces the traditional stopcock valve manifolds found on state-of-the-art radiopharmaceutical synthesis modules by means of a fluidic chip (Figs. 3 and 6) which integrates 70 membrane valves and fluid paths (tubing) as well as 2 reaction chambers into one consumable, enabling channel routing without traditional single fluid bus constraints. The chip can interface to SPE cartridges, different vial formats, sterile filters as well as inlet and outlet tubing employed in radiopharmaceutical syntheses, ensuring compatibility at the *macro-to-micro interface.* The prototype ISAR chips contain two reaction chambers as they were initially developed for complex tracer synthesis like [^18^F]FDG (Frank et al. 2018), and drying and recovery of fluorine-18 in a reproducible way is challenging using alternative microfluidic approaches such as flow reactors, especially when standard vials and SPE cartridges are used. We subsequently employed these prototype reactor-based chips to prepare [^68^Ga]PSMA (Frank et al. 2018).

In combination with real-time valve control that enables gas-pressure driven fluid transport at accuracies comparable to syringe pumps, ISAR combines the benefits of both worlds, by downsizing where it is beneficial (e.g. reduced precursor consumption), while maintaining established output volumes (e.g. 5–25 mL), purification techniques (SPE cartridges), and formulation methods. The flexibility offered by this technology enables parallel fluidic processing. We realized that parallel processing is also well suited for multi-run schemes of simple production processes (e.g. [^13^N]NH_3_), without reuse of contaminated structures and complex cleaning validation (Haka et al. 2017). While such could be possibly be accomplished with homemade valve manifolds, we are unaware of any system capable of handling enough manifolds to offer 9 production lines that are completely separate from each other and enable cGMP synthesis of multiple batches of [^13^N]NH_3_. Moreover, homemade systems are usually controlled by syringe pumps, and not readily fed by single inputs for cyclotron target/water/saline input, meaning footprints can be significantly larger. Contrastingly, ISAR is very compact, uses a single set of reagents for 9 syntheses, and the speed of actuation is higher such that there is no need for syringe pumps. As such, multiple ISAR units can easily be installed in a single min-cell or hot-cell. The flexibility in chip design is an additional advantage of ISAR. Small numbers of new chips can be rapidly accessed for evaluation via rapid prototyping by, for example, 3D printing or milling (Fig. 7). This allows faster implementation of established and new synthesis processes compared with stopcock valve architectures, since the radiosynthesis no longer needs to be customized for the consumable; instead the consumable can now be customized for the process.

With prototype systems built, it was next demonstrated that the ISAR system can be installed at an academic PET Center and used for manufacturing of high quality [^13^N]NH_3_. Installation was quick and simple (< 1 h) and 9 back-to-back batches of [^13^N]NH_3_ were immediately produced without reopening the hot-cell or changing any consumables (Table 3, entry 1). Additional runs with the same parameters were performed (Table 3, entry 2) and confirmed that [^13^N]NH_3_ was obtained at > 96% radiochemical yield based on the input activity. To confirm suitability of [^13^N]NH_3_ prepared on ISAR for clinical use, three additional sub-batches were prepared, and full QC testing was completed (Table 3, entry 3; Table 4). All doses met or exceeded release criteria for clinical application at the University of Michigan, including purity and sterility. Yields were significantly higher than our existing method for producing [^13^N]NH_3_ using Devarda’s alloy (Table 3, entry 4), even accounting for differences in cyclotron parameters (Scott 2012). Moreover, the ability to make 9 sub-batches of [^13^N]NH_3_ from a single consumable, compared with the existing Devarda’s alloy method that requires replacement of consumables and reagents for every sub-batch (Scott 2012), improves our throughput and streamlines daily production of [^13^N]NH_3_ for imaging multiple patients. These improvements in both yield and throughput represent dramatic enhancements to the radiopharmaceutical process resulting from installation of ISAR at the University of Michigan.

**Table 3:** Activity yield of the [^13^N]NH_3_ Radiosyntheses

Entry	Beam	Activity Yield (GBq)	n
1	40 μA for 10 min	7.0 ± 2.0	9
2	40 μA for 10 min	8.9 ± 0.3	8
3^a^	40 μA for 10 min	8.5 ± 0.7	3
4^b^	30 μA for 6 min	2.5 ± 0.2	3
5^c^	40 μA for 10 min	8.8 ± 0.1	3
6^d^	40 μA for 30 min	23.7 ± 0.9	2

^a^ 1st run on chip; ^b^ traditional [^13^N]NH3 production using Devarda’s alloy (see: Scott 2012); ^c^ 2nd run on chip used in entry 3; ^d^ Product was eluted with 8 mL of 0.9% NaCl, Ph. Eur. to simplify handling and QC analysis of the concentrated dose

**Table 4:** QC Data for [^13^N]NH_3_ validation runs. All doses met or exceeded release criteria for clinical application at the University of Michigan

QC Tests	Specifications	Lot 1	Lot 2	Lot 3
Visual Inspection	Clear, colorless, no ppt	Pass	Pass	Pass
Radiochemical-ID	RRT^a^: 0.9–1.1	0.94	0.95	0.97
Radionuclidic-ID	t_1/2_: 9.5–10.5 min	9.99	9.97	10.00
Filter integrity	Bubble point ≥50 psi	53	61	62
Endotoxins	≤43.75 EU/mL	< 2.00	< 2.00	< 2.00
Sterility	No microbial growth	Pass	Pass	Pass
pH value	4.5–7.5	5.0	5.0	5.0
RCP	≥90%	> 99.9%	> 99.9%	> 99.9%

^a^ RRT (Relative Retention Time) = t_R_ [^13^N]NH_3_/t_R_ NH_3_ standard

Although we only anticipate using a chip once for clinical production for the reasons described above, we were next curious from a reliability perspective whether chips could be re-used. Therefore, we investigated whether each fluid path, line and CM cartridge could be used again by rinsing the three channels employed for making the original QC batches (Table 3, entry 3) with 10 mL of sterile water for injection, Ph. Eur. Rinsing was automated and performed without opening the hot-cell. Three additional syntheses of [^13^N]NH_3_ were then performed using the same channels. There was no impact on activity yield (Table 3, entry 5) and QC testing confirmed that these consecutive batches also met or exceeded all the necessary release criteria (data not shown). Lastly, the ISAR system was also tested with higher levels of activity. The irradiation time was increased to 30 min at 40 μA, and the resulting activity was purified and formulated without any anomalies to generate over 20 GBq of [^13^N]NH_3_ (Table 3, entry 6).

In a final demonstration of the suitability of ISAR to produce radiopharmaceuticals for in vivo PET imaging, the heart of a Sprague-Dawley rat was imaged with [^13^N]NH_3_ manufactured using ISAR (Fig. 11). The PET image demonstrated excellent visualization of the heart and good heart-to-background contrast. A region-of-interest was established for the heart and used to generate the associated time-activity curve, which was consistent with previous reports in the literature (Kim et al. 2015). Obtaining the necessary U.S. regulatory approval to use [^13^N]NH_3_ manufactured with ISAR in a clinical setting is underway.

## Conclusion

ISAR represents a potential new paradigm in radiopharmaceutical production capitalizing on decades of synthesizer technology development. Through a new system architecture, ISAR integrates the principles of microfluidics with the standard volumes and consumables established in PET Centers all over the world. A parallel fluidic strategy offers a promising approach for the automated production of radiopharmaceuticals and has been demonstrated for [^13^N]NH_3_, leveraging the ability to create multiple separated fluid paths on a single chip to its full potential. Validation of ISAR for production of other PET radiopharmaceuticals is ongoing. Straightforward compliance with cGMP is possible because of the consumable approach, since the ISAR chips interface with established reagent kits and SPE cartridges, as well as existing aseptic techniques.

## Data Availability

The datasets generated during and/or analyzed during the current study are available from the corresponding author on reasonable request.

## Abbreviations

[^18^F]FDG: [^18^F]fludeoxyglucose
AY: Activity yield
Bq: Becquerels
cGMP: Current good manufacturing practice
COC: Cyclic olefin copolymer
MPI: Myocardial perfusion imaging
PET: Positron emission tomography
QC: Quality control
RCP: Radiochemical purity
SPE: Solid-phase extraction

## Footnotes

1 ISAR is the name of the synthesis module and not an acronym.
